# Plant Calcium Signaling in Response to Potassium Deficiency

**DOI:** 10.3390/ijms19113456

**Published:** 2018-11-03

**Authors:** Xiaoping Wang, Ling Hao, Biping Zhu, Zhonghao Jiang

**Affiliations:** 1 Shenzhen Key Laboratory of Marine Bioresource & Eco-environmental Science, Guangdong Engineering Research Center for Marine Algal Biotechnology, College of Life Sciences and Oceanography, Shenzhen University, Shenzhen 518060, China; wangxp@szu.edu.cn (X.W.); haoling2017@szu.edu.cn (L.H.); Musyfreak@hotmail.com (B.Z.); 2 Key Laboratory of Optoelectronic Devices and Systems of Ministry of Education and Guangdong Province, College of Optoelectronic Engineering, Shenzhen University, Shenzhen 518060, China

**Keywords:** calcium signaling, potassium deficiency, calcium sensors, CBLs, CIPKs

## Abstract

Potassium (K^+^) is an essential macronutrient of living cells and is the most abundant cation in the cytosol. K^+^ plays a role in several physiological processes that support plant growth and development. However, soil K^+^ availability is very low and variable, which leads to severe reductions in plant growth and yield. Various K^+^ shortage-activated signaling cascades exist. Among these, calcium signaling is the most important signaling system within plant cells. This review is focused on the possible roles of calcium signaling in plant responses to low-K^+^ stress. In plants, intracellular calcium levels are first altered in response to K^+^ deficiency, resulting in calcium signatures that exhibit temporal and spatial features. In addition, calcium channels located within the root epidermis and root hair zone can then be activated by hyperpolarization of plasma membrane (PM) in response to low-K^+^ stress. Afterward, calcium sensors, including calmodulin (CaM), CaM-like protein (CML), calcium-dependent protein kinase (CDPK), and calcineurin B-like protein (CBL), can act in the sensing of K^+^ deprivation. In particular, the important components regarding CBL/CBL-interacting protein kinase (CBL/CIPK) complexes-involved in plant responses to K^+^ deficiency are also discussed.

## 1. Introduction

**Figure 1:**
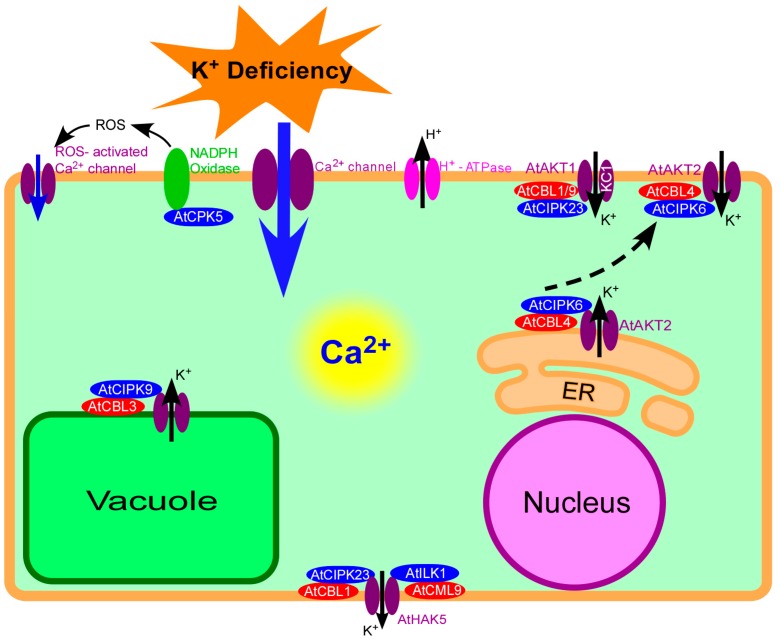
Calcium signaling in *Arabidopsis* response to K^+^ deficiency. Plants can perceive external K^+^ deficiency and generate K^+^ deficiency signals in plant cells. The signal Ca^2+^ can be transduced in cytosol, and eventually regulate the downstream targets at the transcriptional and posttranslational levels. ER: Endoplasmic Reticulum.

Potassium (K^+^) is an essential macronutrient and is essential for plant growth and development [1]. K^+^ is associated with or involved in several physiological processes that support plant growth and development, such as photosynthesis, enzyme activation, osmoregulation, electrical neutralization, pH and ion homeostasis, anion-cation balance, membrane electrical potential, protein and starch synthesis, sugar and nutrient transport, and stomatal movement [2]. K^+^ also plays a major role in enhancing the tolerance of plants to various stresses [3,4]. The concentrations of K^+^ in the soil solution range from only 0.1–1 mM, and can be much lower at the root surface due to local depletion [5]. K^+^ deficiency in most arable fields is limiting for optimal plant growth [6,7]. K^+^ deprivation leads to a strong increase in chlorophyll degradation; K^+^ deficiency-related symptoms include brown scorching and curling of leaf tips, as well as interveinal chlorosis [8]. Reduced leaf area under K^+^ deficiency has also been reported [9,10]. In addition, K^+^ deficiency affects root development, as primary root growth is negatively affected [11,12]. Various K^+^ shortage-activated signaling cascades exist; these cascades involve reactive oxygen species (ROS) [13], phytohormones (ethylene, auxin, and jasmonic acid) [14,15], calcium [16], and phosphatidic acid [17]. Among these signaling cascades, calcium signaling is the most important signaling system within plant cells. In this review, the possible roles of calcium signaling in plant responses to low-K^+^ stress are discussed (Figure 1).

## 2. Molecular Mechanisms of Calcium Signaling Involved in Plant Responses to K^+^ Deficiency

### 2.1. Generation of Calcium in Response to K^+^ Deficiency

The concentration and distribution of cytosolic free calcium form the basis of calcium signaling. Under normal conditions, levels of cytosolic free calcium are low, but some organelles, including the vacuole, endoplasmic reticulum, mitochondria and so on, contain high concentrations of calcium, henceforth referred to as the calcium pool. Elevations in intracellular calcium [Ca^2+^]_i_ have been recorded in the responses of both lower and higher plants to a wide variety of both biotic and abiotic stimuli [18,19]. In plants, [Ca^2+^]_i_ levels that are altered in response to multiple abiotic stresses result in calcium signatures that exhibit temporal and spatial features [20,21,22]. These calcium signatures can take the form of single calcium transients [23,24], oscillations [25,26,27], or repeated spikes [28,29]. Alterations to cytosolic calcium signals can be perceived by calcium sensors, which can result in a series of downstream responses, such as protein modification and transcriptional regulation [30,31,32,33,34,35,36,37,38]. Calcium sensors in *Arabidopsis* root are involved in both K^+^ uptake and responses to K^+^ deficiency. Low K^+^ induces [Ca^2+^] to increase in *Arabidopsis* guard cells [39] and in the pollen tubes [40]. The results of a recent study revealed that K^+^ deficiency triggers two successive but distinct calcium signals in roots, and that those two signals exhibit spatial and temporal specificity [16]. Calcium flows into or out of the cytoplasm via calcium channels located within the plasma membrane and endomembrane system [18,41,42]. Most calcium channels are nonselective for ions [41,43]. In plants, these calcium channels mainly include nonspecific cation channels located within the cell membrane [43,44], including members of the cyclic nucleotide-gated channel (CNGC) family [26,45,46,47] and the glutamate receptor channel (GLR) family [48,49,50], hyperosmolality-gated calcium-permeable channels [51,52], and annexins proteins [53], and mechanosensitive channels (MCAs) [54], as well as two-pore calcium channels (TPCs) [55,56] located in the vacuolar membrane. However, the mechanisms of their action remain unclear.

### 2.2. Initial Sensing of K^+^ Deficiency by Calcium Channels

In plants, although some calcium channel activation occurs via depolarization [57,58,59], most channels operate at highly-negative membrane voltages, and are often described as hyperpolarization-activated calcium channels (HACCs) [41,53,60,61,62,63]. After low-K^+^ stress, calcium channels located within the root epidermis and root hair zone can be activated by hyperpolarization of the PM [60,64]. Calcium increases in the cytosol can activate additional calcium channels located within the inner membrane, thereby causing the calcium pool to release calcium. For instance, two-pore channel 1 (TPC1), which is a voltage-gated channel and is located within the vacuolar membrane, is involved in the influx of calcium to the cytoplasm from the vacuole [55]. In addition, the low-K^+^-inducing [Ca^2+^]_i_ increase is also mediated by ROS-activated calcium channels in the PM [61]. ROS are important signaling molecules that mediate many physiological stimuli and lead to the generation of [Ca^2+^]_i_ signals under stress [65,66]; increases in ROS are induced by K^+^ deficiency [67,68]. Elevated [Ca^2+^]_i_ can induce NADPH oxidase-mediated production of ROS, which in turn activates calcium-permeable ion channels, thereby resulting in further calcium influx [62,69]. Additionally, ROS have been also suggested to participate in long distance signaling with calcium, and are likely involved in generating calcium waves [70,71,72,73].

### 2.3. Calcium Sensors Involved in the Sensing of K^+^ Deprivation

How plant cells sense transient increases in [Ca^2+^]_i_ in response to low-K^+^ stress is ambiguous. Calcium signals are likely perceived by calcium sensors, decoded, and further transduced. Different calcium sensors must exist in the cytoplasm of plant cells so that different calcium signals can be recognized. In higher plants, putative calcium sensors include CaM, CML, CBL, and CDPK, which are derived mainly from four gene families [18,74,75,76,77] and which are collectively encoded by approximately 100 genes in the *Arabidopsis* genome [30]. Calcium sensors can be divided into two types: sensor responders and sensor relays. Sensor responders, such as CDPK, usually undergo a calcium-induced conformational change that alters the protein’s own structure and activity. On the other hand, sensor relays, such as CaM, CML, and CBL, lack responder domains; these sensors first combine with calcium and subsequently undergo a conformational change that is relayed to an interacting partner. The interacting partner then responds with some change in its enzyme activity or structure. The two types of calcium sensors differ by their action: sensor responders function via intramolecular interactions (e.g., CDPK), whereas sensor relays function via biomolecular interactions (e.g., CBL interact with CIPK) [78,79].

Calcium sensor responders mainly consist of CDPKs. CDPKs, which are a class of calcium-dependent protein kinases first discovered in plants, are serine/threonine protein kinases, and are probably the best-studied protein kinases involved in signal transduction in plants [76,77,80]. CDPKs are monomeric proteins with a molecular mass of 40 to 90 kDa, and consist of five domains: an N-terminal variable domain, a protein kinase catalytic domain, an autoinhibitory domain, a regulatory domain, and a C-terminal domain of variable length [75,76,77]. The autoinhibitory domain contains a pseudo-substrate sequence that can interact with the active site and inhibit its activity. Once a calcium signal is generated, the autoinhibition of CDPKs is relieved; therefore, the CDPKs become activated, and the activated CDPKs can subsequently phosphorylate target enzymes or molecules, leading to physiological responses [81,82,83]. In addition to CDPKs, Ca^2+^/CaM-regulated kinases and chimeric Ca^2+^ and Ca^2+^/CaM regulated kinases (CCaMKs) are also members of this family of Ca^2+^-regulated protein kinases [30]. CPK10 is involved in the Ca^2+^-dependent inhibition of K^+^_in_ channels in guard cells [84]. Together, CPK11 and CPK24 mediate Ca^2+^-dependent inhibition of the activity of shaker pollen inward K^+^ channels (SPIK/AKT6) in pollen tubes, further increasing our understanding of the CDPK-mediated regulatory mechanisms of K^+^ channels [40]. CPK13 specifically inhibits guard cell-expressed KAT2 and KAT1 shaker K^+^ channels [85]. The results of a recent study [86] showed that two *CDPKs* are up-regulated in tobacco seedlings under low-K^+^ stress, whereas *OsCPK9* and *OsCCaMK1* are down-regulated in rice root responses to K^+^ deficiency [87].

Calcium sensor relays mainly consist of CaMs and CML [88]; CaM is an important member of this class of calcium sensor proteins. Plant CaMs are small, acidic proteins with a molecular weight ranging from 16.7–16.8 kDa; CaM contains 4 EF-hand repeated domains that bind 4 individual Ca^2+^ ions. In addition, the four binding sites display cooperativity in Ca^2+^ binding; as such, unbound CaM is fully opened [89,90]. CaM binding with Ca^2+^ leads to a conformational change. This change exposes the hydrophobic surface of CaM, which helps to interact with target proteins in a Ca^2+^-dependent manner, altering their activities. CaM binding with Ca^2+^ forms an activated Ca^2+^-CaM complex, and the binding of this complex to target enzymes leads to their activation [91,92]. CaM has no catalytic activity of its own, but upon binding with Ca^2+^, CAM modulates the activities of several enzymes and non-enzymic proteins involved in a variety of cellular processes [93,94,95]. CML also acts in signaling ‘cross-talk’ and is involved in the co-ordination of plant responses to biotic and abiotic stresses [21,96,97]. CML25 is an important transducer involved in the Ca^2+^ ions-mediated regulation of K^+^ influx [98]. Ma et al. (2012) [87] reported that the expression levels of the calcium sensor protein genes *OsCML1*, *OsCML18*, *OsCML20*, and *OsCML31* are up-regulated in rice under low-K^+^ stress. Several genes that code for members of CML families are differentially expressed in tobacco seedlings under low-K^+^ stress [99]. In addition, a Raf-like MAPKK kinase (AtILK1) directly interacts with AtHAK5 in conjunction with the AtCML9, promoting AtHAK5 accumulation on the membrane [100]. These results indicate that calcium sensor proteins may play vital roles in connecting calcium signaling and downstream target proteins during plant responses to K^+^ deficiency.

Another important member of the calcium sensor relays is CBL, which, like CaM and CML, has been shown to lack responder domains [101]. CBLs and CBL-interacting protein kinases (CIPKs) often form the CBL/CIPK complexes, perceiving calcium signals and relaying the signals to downstream responses in plants under low-K^+^ stress [31,32,33,34,35,37,102,103,104,105].

## 3. Role of the CBL/CIPK Complex in Response to K^+^ Deficiency

### 3.1. CBL Proteins

CBL proteins, which are involved in the salt-overly-sensitive (SOS) pathway of salt stress signal transduction, were first identified in *Arabidopsis* [101]. CBL proteins in plants share significant sequence similarities with the calcineurin B subunit in yeast and the neuronal calcium sensors in animals [74,101,106]. In *Arabidopsis*, CBLs are encoded by at least 10 members of a multigene family [106,107,108]. Each CBL protein harbors four EF-hand motifs that facilitate Ca^2+^ binding; in all CBL proteins, these motifs are arranged in fixed spacing. Therefore, the weights of CBLs are nearly equal (23–26 kD); the N-terminal and C-terminal domains account for the differences in CBL weights. Unlike in other species, in *Arabidopsis*, the first CBL EF-hand domain consists of an unconventional 14 amino acids, not 12 amino acids; this *Arabidopsis* CBL EF-hand domain likely lacks the normal amino acid numbers required for Ca^2+^ binding [107,109]. The crystal structures of AtCBL2 and AtCBL4 indicate that two calcium ions are coordinated in the first and fourth EF-hand motifs despite the presence of two additional amino acids [110,111]. Sequence variations in EF-hand motifs most likely control the overall Ca^2+^-binding affinity of individual CBL proteins. This phenomenon may explain why plants can decode different calcium signals [109,112,113,114,115,116].

With the exception of CBL10, whose hydrophobic N-terminal region is a special transmembrane domain, other CBLs can be classified into one of two categories based on variations in their N-terminal domain. The first category represents CBL proteins with a short N-terminal region, which consist of 27–32 amino acids. Examples in this category include CBL1, CBL4, CBL5, CBL8, and CBL9; other CBL proteins, with exception of CBL8, all have a MGXXX(S/T) consensus sequence for N-myristoylation [117]. The second category represents CBL proteins with an extended N-terminal region, which consist of 41–43 amino acids. Examples in this category include CBL2, CBL3, and CBL6, which lack recognizable lipid modifications [117]. Sequence comparisons place CBL7 within this category; therefore, the CBL7 protein appears to have lost its N-terminal extension during evolution [106,112].

CBL proteins were first identified in model plant *Arabidopsis* [74,101]; subsequent bioinformatics analysis has revealed that these kinds of proteins also exist in other species. The *Oryza sativa* (rice) genome contains 10 genes that encode CBL proteins [108], and the *Populus trichocarpa* (poplar) genome also contains 10 genes that encode CBL proteins [118]. The fully-sequenced genomes of the dicotyledonous plant *Vitis vinifera* (grape) and the monocotyledonous plant *Sorghum bicolor* (sorghum) have been analyzed in attempt to detect the presence of CBLs; these analyses revealed 8 CBLs in grape and 6 CBLs in sorghum [112]. *Gossipium raimondii* (cotton) was found to contain the highest number of CBL genes (13) among the 38 plant species analyzed [119]. And 19 members of the *BrrCBL* genes were identified in *Brassica rapa* var. *rapa* (turnip) [120]. A comparative analysis of CBLs from all these species further supports the classification of these proteins according to their N-terminal domain [112]. An analysis of the genomic sequences of algae and nonvascular plants revealed that the genome of the moss *Physcomitrella patens* encodes 4 CBL proteins, and that the genome of the fern *Selaginella moellendorffii* also encodes 4 CBL proteins. In addition, one CBL protein was identified in the green alga *Chlorella* sp., as well as in the genome of the smallest known free-living eukaryotic alga, *Ostreococcus tauri* [121]. In general, these studies of lower plants have enabled us to address the general evolutionary aspects of this signaling network [112].

### 3.2. CBL-Interacting Protein Kinases (CIPKs)

Protein kinases that specifically interact with CBL proteins have been identified; these kinases are referred to as CIPKs, which were mentioned earlier with salt-overly-sensitive 2 (SOS2), SOS3-interacting proteins (SIPs), and protein kinase S (PKS) [122,123,124]. All of these CIPKs share a typical two-domain structure comprising an N-terminal kinase domain and a C-terminal catalytic domain, which are separated by a junction domain [109,112]. Sucrose non-fermenting 1 (SNF1)-related protein kinases (SnRKs) are important kinases in plants, and exhibit high sequence homology to metabolic regulators found in mammals (5′-AMP-activated protein kinases [AMPKs]) and in yeast (SNF1); the three combined kinases form the protein kinase superfamily. Amino acid sequence identification and expression pattern analyses have revealed that the SnRK family of protein kinases can be categorized into three classes: SnRK1, SnRK2, and SnRK3 [125,126,127]. *Arabidopsis* CIPKs constitute a kind of serine-threonine kinase, and have a highly-conserved N-terminal catalytic domain; these proteins have been classified as SnRK3s [125,128].

The N-terminal domain of a protein kinase contains a conserved activation loop. Assays of mutants revealed that the Thr168, Ser156, or Tyr175 to Asp change in the activation loop of CIPK24/SOS2 protein kinase [129,130,131], the Thr178 to Asp change in the activation loop of CIPK9/PKS6 [132], the Thr161 to Asp change in the activation loop of CIPK8/PKS11 [133], and the Thr183 to Asp change in the activation loop of CIPK3 [134] can cause strong activation of CIPK protein kinases, even in the absence of Ca^2+^ or CBLs. Thus, these several conserved amino acid residues may be phosphorylation sites of CIPK protein kinases, whose phosphorylation results in the activation of CIPK [112]. Furthermore, an additional phosphorylation site (Ser 228) has been identified as a target of autophosphorylation activity in the C-terminal region of the CIPK24/SOS2 kinase domain [135].

Within the otherwise divergent C-terminal regulatory domain, CIPKs have a conserved domain, a NAF motif, and a 24-aminoacid domain with the conserved amino acids N, A, and F that are required for the CBL-CIPK interactions; the NAF motif is also referred to as a FISL motif because of the complete conservation of the six amino acid residues A, F, I, S, L, and F. The NAF/FISL motif of CIPKs is sufficient for mediating protein interactions with all CBL proteins [128,129,136,137]. The NAF/FISL motif is also necessary and sufficient for keeping CIPKs inactive, and serves as an autoinhibitory domain; removal of the NAF/FISL domain can increase the activity of CIPKs [129,132].

Another important functional domain is the protein phosphatase interaction (PPI) motif in the C-terminal region of CIPKs; this motif is adjacent to the NAF/FISL motif. The PPI motif consists of 37 amino acid residues, and was first identified in CIPK24/SOS2, which is necessary and sufficient for interaction with ABA-INSENSITIVE 2 (ABI2) [138]. The PPI motif is conserved in CIPK protein kinases; all of these motifs interact with 2C-type protein phosphatase (PP2C) [34,128,138,139]. In plants, PP2C is a strong negative regulator of the stress-activated, mitogen-activated protein kinase (MAPK) pathway, which is involved in plant responses to abiotic stresses and growth regulation [140,141,142]. PP2C interaction with CIPK results in the complete replacement of the combination between CBL proteins and the NAF domain or a portion of the PPI domain of CIPKs [112]. The dissociation of CBL proteins from the NAF domain of CIPK prevents the autophosphorylation of CIPK, thereby transforming the kinase into an inactive state [129,132].

### 3.3. Involvement of the CBL/CIPK Complex in the Sensing of K^+^ Deficiency

**Figure 2:**
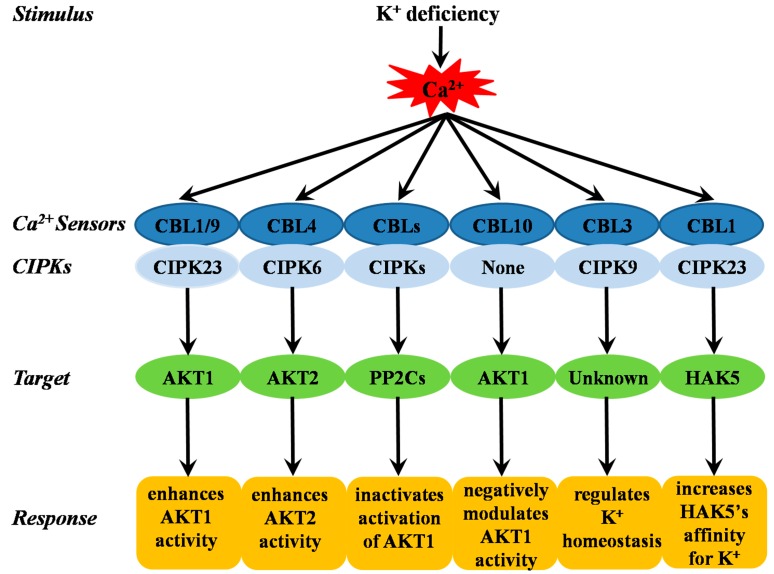
The summary of CBL-CIPK complex involved in response to K^+^ deficiency.

Some CBL protein family members that interact with CIPKs function in plant responses to K^+^ deficiency (Figure 2) [31,32,33,34,35,37,102,103,104,105]. The first CBL proteins identified to be involved in the K^+^ deficiency response were the PM-localized calcium sensors CBL1 and CBL9; these sensors interact with the cytoplasm-localized Ser/Thr kinase CIPK23, and recruit it to the root cell PM, where the complex subsequently phosphorylates AKT1 [31,102,143]. AKT1, a shaker inward K^+^ channel [144,145,146], is considered a major component involved in K^+^ uptake in *Arabidopsis* root cells under low-K^+^ conditions [147,148,149]. In addition, AtKC1, a K^+^ channel regulatory subunit that negatively modulates many inward K^+^ channels, interacts with AtAKT1, forming an AtAKT1-AtKC1 heteromeric channel, and modulates AtAKT1 activity together with AtCIPK23, to synergistically regulate AtAKT1-mediated low-K^+^ stress responses [150,151]. Another CBL protein, CBL10, was recently shown to be a negative regulator of the AKT1 channel. CBL10 may compete with CIPK23 for binding to AKT1, and CBL10 interacts directly with the AKT1 channel and inhibits AKT1-mediated K^+^ flux into the cytoplasm. In *Arabidopsis*, this inhibition ultimately maintains K^+^ homeostasis under ion stress conditions in a CIPK-independent manner [35]. The calcium sensor CBL4, together with the interacting protein kinase CIPK6, modulates the activity and PM targeting of the K^+^ channel AKT2 in *Arabidopsis*; CBL4 in conjunction with CIPK6 mediates the translocation of AKT2 from the endoplasmic reticulum membrane to the PM in a kinase-interaction-dependent but phosphorylation-independent manner in plant cells, and enhances AKT2 activity in oocytes [33]. AKT2 is unique among the nine shaker-type K^+^ channel subunits expressed in *Arabidopsis*, because AKT2 exhibits weak inward-rectifying activity in oocytes [152,153]; several studies have suggested that this channel is regulated by unknown protein kinases and by the protein phosphatase PP2CA [154,155,156]. A recent study demonstrated that CBL3 and CIPK9 work together and function in K^+^ homeostasis under low-K^+^ stress; this complex mediates the regulation of putative tonoplast-localized outward K^+^ channels [104]. CIPK9 is the CIPK family member which is most similar to CIPK23, and CIPK9 loss of function results in a phenotype that is tolerant to K^+^ deficiency conditions [31]; in contrast, CIPK9 overexpression lines are sensitive to K^+^ deficiency stress. Furthermore, because K^+^ deficiency symptoms first appear in relatively old leaves, CIPK9 may be involved in K^+^ reallocation from older leaves to the younger leaves during K^+^ deficiency [32,157]. In addition, the AtCBL1/AtCIPK23 complex can phosphorylate AtHAK5, which is a KT/KUP/HAK-type transporter whose expression occurs mainly in the roots [37]. The transcription of *HAK5* is induced by K^+^ deficiency via the transcription factor RAP2.11 [147,158], which is considered to function predominantly in the uptake of K^+^ from the soil [37,145,147,159,160,161].

These CBLs-CIPKs-AKT1/AKT2/HAK5 pathways are important mechanisms in the response to low-K^+^ stress in *Arabidopsis*. Similar mechanisms have also been identified in other plant species. The OsCBL1-OsCIPK23-OsAKT1 pathway was identified in rice [36]. Rice OsCIPKs show high amino acid sequence similarity to *Arabidopsis* CIPKs; eight OsCIPK genes *(OsCIPK2*, *6*, *9, 10*, *14*, *15*, *23*, and *26*) are upregulated under low-K^+^ stress, whereas two OsCIPK genes (*OsCIPK29* and *31*) are down-regulated [108]. Another study demonstrated that the AtCBL9/AtCIPK23 kinase complex activates DmKT1, which has been identified as a K^+^-selective channel of voltage-dependent high capacity and low affinity; the first proton-driven high-affinity K^+^ transporter with weak selectivity (DmHAK5) is also activated by the same kinase complex [38].

Furthermore, CBL/CIPK (CIPK6, CIPK16, and CIPK23) complexes can interact with the AKT1-interacting PP2CA (AIP1) and AIP1 homologue (AIP1H), both of which are protein phosphatase PP2Cs, via the direct binding of the kinase domain of CIPKs to indirectly deactivate AKT1 by inhibiting phosphorylation. Several CBLs have been reported to interact with and inhibit the activity of PP2Cs, thereby enhancing CIPK-induced AKT1 activation; this phenomenon forms a kinase/phosphatase partnership that enables AKT1 activity to be switched on and off [34,103]. In addition, AtPP2CA can interact physically and functionally with AKT2, and can inhibit AKT2-mediated K^+^ currents via the direct phosphorylation of AKT2 [154].

## 4. Conclusions and Perspectives

K^+^ is an essential macronutrient and is associated with or involved in several physiological processes supporting plant growth and development, such as photosynthesis, enzyme activation, osmoregulation, electrical neutralization, pH and ion homeostasis, anion-cation balance, membrane electrical potential, protein and starch synthesis, sugar and nutrient transport, and stomatal movements. K^+^ deprivation leads to a strong increase in chlorophyll degradation. Various K^+^ shortage-activated signaling cascades exist; these cascades involve ROS, phytohormones, calcium, and phosphatidic acid. Among these signaling cascades, calcium signaling is the most important signaling system in plant cells. Thus far, our knowledge on the molecular mechanisms of calcium signaling in plant responses to K^+^ deficiency is still limited.

Calcium plays a critical role in plant responses to low-K^+^ stress. Although tremendous progress has been made in understanding plant responses to low-K^+^ stress, one important question that remains unanswered is how calcium as a messenger can relay information that distinguishes different extracellular signals, triggering different processes and specific responses in cells. The results of the previous studies suggest that K^+^ deficiency can induce changes in intracellular calcium levels that exhibit temporal and spatial features. The decoding of calcium signatures in plant cells might depend largely on the presence of various calcium sensors, including CaM, CML, CDPK, and CBL, as well as their targets. In particular, the CBL-CIPK signaling system is a central and critical signaling system for decoding calcium signatures and for translating those calcium signatures into downstream responses to K^+^ deficiency.

Over the last two decades, the extensive genomic, genetic, and molecular physiological studies have begun to shed light on the transport regulation and signaling mechanisms of plant responses to K^+^ deficiency. Further identification of important calcium signaling components involved in plant responses to K^+^ deficiency is important. Detailed functional characterization of these calcium-signaling components is also needed to elucidate the complex network of plant signaling in response to K^+^ deficiency. Even though many K^+^ transporters and channels in higher plants have been functionally characterized, specific K^+^ sensors remain unknown. Thus, future investigations should give attention to further functional characterization of K^+^ sensors and to the regulatory mechanisms of these sensors. Given that K^+^ deficiency could induce [Ca^2+^]_i_ to increase in *Arabidopsis* guard cells and in the pollen tubes or triggers calcium signals in *Arabidopsis* roots, the perception-defective of K^+^ deficiency mutants are likely to be screened via detection of the plant calcium signal.
